# A porous collagen-based hydrogel and implantation method for corneal stromal regeneration and sustained local drug delivery

**DOI:** 10.1038/s41598-020-73730-9

**Published:** 2020-10-09

**Authors:** Maria Xeroudaki, Muthukumar Thangavelu, Anton Lennikov, Anjula Ratnayake, Jovana Bisevac, Goran Petrovski, Per Fagerholm, Mehrdad Rafat, Neil Lagali

**Affiliations:** 1 Department of Ophthalmology, Institute for Biomedical and Clinical Sciences, Linköping University, 58183 Linköping, Sweden; 2 Department of Biomedical Engineering, Linköping University, 58185 Linköping, Sweden; 3 LinkoCare Life Sciences AB, 58330 Linköping, Sweden; 4 Department of Ophthalmology, Centre for Eye Research, Oslo University Hospital and Institute of Clinical Medicine, University of Oslo, Oslo, Norway; 5 Department of Ophthalmology, Sørlandet Hospital Arendal, Arendal, Norway

**Keywords:** Biomaterials, Implants, Translational research, Corneal diseases

## Abstract

Biomaterials designed to replace the diseased cornea could be used to treat corneal blindness where human donor tissue is in short supply, but challenges are the integration of biomaterials with host tissue and cells, avoiding a rapid material degradation and maintaining corneal transparency. Additionally, implantation surgery often triggers an aggressive wound healing response that can lead to corneal thinning and opacity. Here, we report a collagen-based hydrogel with transparency and mechanical properties suitable for replacing a substantial portion of a damaged or diseased corneal stroma. The porous hydrogel permitted migration and population by host cells while maintaining transparency and thickness six months after surgical implantation in an in vivo model of human corneal surgery. With a novel hybrid surgical implantation technique inspired by LASIK refractive surgery, rapid wound healing occurred around implants to maintain biomaterial integrity, transparency and function. Host stromal cell repopulation and regeneration of host epithelium and nerves were observed, as necessary steps towards corneal regeneration. Finally, as a proof-of-principle, the hydrogel loaded with a neuroregenerative drug achieved sustained slow-release drug delivery in vitro. The proposed hydrogel and novel implantation technique together represent a therapeutic approach with translational potential for replacing and regenerating diseased corneal stromal tissue.

## Introduction

The cornea is the most commonly transplanted tissue worldwide, but an inadequate supply of corneal tissue results in millions of cases of untreated corneal blindness^1^. Indications for corneal transplantation include a range of diseases from degenerative and dystrophic conditions to infectious and inflammatory corneal disorders, where the cornea, and particularly the corneal stroma, loses its transparency. For instance, keratoconus, a disease of progressive thinning of the corneal stroma, is the leading indication for corneal transplantation in Australia, the Middle East, Africa and South America and the second leading indication globally^1,2^. The limited global availability of corneal tissue, however, has spurred the development of alternative approaches based on tissue engineering and regenerative medicine (reviewed in^3,4^).

Conventional full-thickness corneal transplantation (penetrating keratoplasty) has long been the standard method of corneal replacement, but recently this technique has been complemented by newer methods selectively replacing a specific corneal layer. These so-called lamellar keratoplasty techniques have the advantage of being less invasive, as only the diseased part of the cornea is replaced while leaving the surrounding healthy tissue intact. Examples of such techniques are anterior lamellar keratoplasty^5^ and endothelial keratoplasty^6^ that provide improved visual outcomes, higher graft survival rates, and less postoperative complications compared to full-thickness penetrating keratoplasty.

In cases where the indication for vision-restoring surgery is corneal stromal pathology such as keratoconus, corneal scarring or dystrophies, the least invasive procedure is to replace only the affected stromal tissue^3^. Although approaches such as re-use of donor-derived refractive surgical stromal lenticules (obtained using small-incision lenticule extraction, or SMILE) to treat stromal disease such as keratoconus^7^ or corneal perforation^8^ are gaining popularity, the human donor-sourced material is still scarce, and is only available in limited thickness and diameter. Alternatively, by bioengineering corneal tissue from raw starting materials, one avoids reliance on donor tissue and the engineered tissue can be manufactured to desired specifications. In a phase I human clinical study conducted in Sweden, engineered recombinant human collagen-based implants were used as anterior lamellar grafts in ten patients with advanced keratoconus or stromal scarring^9^. The anterior lamellar grafting, however, was moderately invasive and required removal of native corneal epithelium and stroma. Moreover, the anterior grafts were secured with overlying sutures as they had only a fraction of the strength required^10^ for direct suturing through the material, as is commonly done with traditional donor tissue. Although 4 years after implantation the implanted materials were stably integrated without rejection, vision recovery was limited in some patients due to surgical suture-induced delayed re-epithelialization of implants and ocular surface irregularities^11^. Host cells did not appear to repopulate the implanted material to induce a stromal regenerative response^3,11^, while corneal nerve regeneration, critical for wound healing and long-term corneal homeostasis, was achieved to the extent expected following standard transplantation but not to normal levels^11^.

To address these limitations and acknowledging the limited global supply of recombinant human collagen, we recently developed a bioengineered porcine collagen (BPC) platform based on high-purity medical-grade collagen extracted from porcine skin, manufactured to withstand surgical implantation in the eye. Here we report on material morphology, transparency, mechanical properties, and biocompatibility using cell culture, subcutaneous implantation assays, and implantation in rabbit models. Prior experimental surgery using similar materials^12^ tested small-diameter, thin implants replacing a minimal proportion of the corneal stroma. Here, we aimed to evaluate the BPC implants for the first time in an in vivo model mimicking corneal surgery in patients, using implants with a diameter and thickness resembling human corneal surgery.

To facilitate ease of implantation in a clinical setting, two ophthalmic femtosecond laser-assisted surgical techniques were used to enable these thicker and larger implants to replace a greater proportion of the corneal stroma, as would be required to treat corneal diseases. We recently introduced the use of an ophthalmic femtosecond laser for intrastromal implantation of bioengineered corneal substitutes in a surgical procedure termed FLISK^12,13^ in order to preserve the corneal epithelium and its associated nerves, and avoid the need for surgical sutures coming in contact with implanted materials. Thus the risk of suture-related complications including induced astigmatism and delayed epithelialization are minimized. Here we compare the standard anterior lamellar keratoplasty (ALK) technique of implantation and suturing to a new intrastromal method, using a hybrid LASIK-type flap-based surgical approach (Flap-ALK) that can be applied even manually where a femtosecond laser is unavailable. The flap-ALK technique preserves the epithelium and a portion of the underlying stroma and nerves, thereby reducing the wound extent relative to standard ALK. These surgical techniques were evaluated with the BPC implants in terms of postoperative corneal structure and transparency, inflammatory response, and the effect on nerve and cell repopulation of the cornea.

Finally, the ability of BPC implants to actively promote regeneration was investigated in a first proof-of-concept experiment. After loading the porous BPC implants with recombinant nerve growth factor, a regenerative drug, its sustained release dynamics were measured in vitro*.*

## Materials and methods

### Fabrication of bioengineered porcine collagen (BPC) hydrogels

We have previously reported on the fabrication of bioengineered corneal implants using porcine collagen^14,15^. We have also reported the fabrication method for 100 μm thin and flat BPC implants^12^. Here, we report on implants made from high-purity, medical-grade porcine collagen (type-I atelo-collagen) crosslinked by 1-[3-(Dimethylamino) propyl]-3-ethylcarbodiimide (EDC) and N-hydroxysuccinimide (NHS). The water-soluble, zero-length crosslinkers EDC and NHS were added to an 18% collagen solution and after mixing the resulting solution, a compression molding method was used. For this study, curved implants of pre-defined thicknesses were prepared, with final hydrogels having thicknesses of 280 μm and 440 µm. The thinner gels were designed for intra-stromal implantation while the thicker versions were to be used for ALK. To produce the implants, different molds were used for each thickness, with all molds having the shape of curved contact lenses to mimic the corneal curvature.

### Surface microstructure and optical characterization of BPC hydrogels

To characterize the surface morphology, BPC hydrogels were removed from the PBS solution, immediately frozen to − 80 °C overnight, and lyophilized for 5–6 h. Implants were then fixed for scanning electron microscopy (SEM) analysis on a sample holder and sputter-coated with gold layers for 60 s at 0.1 bar vacuum pressure. SEM images were captured at different magnifications (SEM Model S-2250 N; Hitachi). Optical transmission measurements were performed using hydrated implants immersed in PBS at room temperature. Light transmission of the corneas was measured over a full range of UV and visible light spectrum from 200 to 700 nm at room temperature using a High Performance USB4000 UV–Vis Spectrophotometer. Thick hydrogels (e.g. 500 μm in thickness) were used for the light transmission measurements to enable comparison with human corneas.

### Biocompatibility of BPCs with human corneal epithelial cells

All tissue collections were in the accordance with the Guidelines of the Helsinki Declaration. Human corneal epithelial cells (HCEC) harvested from human donor corneas were obtained from the Eye Bank at the Center for Eye Research, Department of Ophthalmology, Oslo University Hospital (Regional Committees for Medical and Health Research Ethics, Norway ethical permit no. REK 1.2007.1099). Primary HCEC from two different donors (ID 16188 and 16200) were biopsied and treated with Dispase II (2.4 U/ml, Roche Diagnostics GMBH, Mannheim, Germany) for 10 min at 37ºC, blocked with fetal bovine serum (Sigma-Aldrich) and plated on 6-well plates (Corning Inc.) and maintained in a complex medium containing DMEM/F12 medium (Thermo Fisher Scientific, Waltham, MA, USA), 100 U/ml penicillin, 100 μg/ml streptomycin, 1.25 μg/ml amphotericin B, 5% FBS, 2 ng/ml human epidermal growth factor, 5 μg/ml insulin–transferrin–sodium selenite, 15 μM hydrocortisone, 0.5% dimethylsulfoxide, and 30 ng/ml cholera toxin A (all from Sigma Aldrich) in a humidified 5% CO_2_ incubator at 37 °C. After the cells growing out of biopsies reached confluence they were harvested and seeded onto 96 well cell culture plates (1 × 105 cells/well) on top of hydrogels or plate bottom (control). Cell culture media were changed every 3 days, and cell cultures continued until 14 days. After 14 days of incubation, (3-(4,5-Dimethylthiazol-2-yl)-2,5-Diphenyltetrazolium Bromide) MTT assay (% cell viability) and Live/Dead Viability/Cytotoxicity assay (Invitrogen) was performed in triplicate. Percentage of viable cells was determined by photometric measurements of the blue Formazan product at 570 nm, and calculated as the ratio of absorbance of BPC-grown cells to absorbance of the control cells, with each absorbance corrected by the reading of a blank sample. Cells stained for Live/Dead viability were photographed with a Nikon smz1500 microscope.

### *Ex-vivo* suture test

Prior to in vivo implantation, a 7.25 mm diameter trephine punch (Barron vacuum corneal trephine, Katena Products Inc., NJ, USA) was used to cut circular buttons of 440 µm thick curved BPC implants, while a 7.00 mm diameter trephine punch was used to cut circular buttons of 280 μm thick curved BPC implants. For the *ex-vivo* suture test, suturing of the BPC was tested by implanting the 440 µm-thick specimens as a full thickness graft into whole enucleated porcine eyes (obtained from a local meat processing plant). After placing a porcine eye into a vacuum holder, a full thickness corneal button was removed from the native cornea by manual trephination. A BPC implant 440 µm thick was then sutured into the recipient bed using 8 interrupted circumferential 10-0 nylon sutures while keeping the construct hydrated using Optisol corneal storage medium. The number of sutures placed and the number of knots successfully buried, tearing of the biomaterial, and tissue distortion were recorded. The microstructure was subsequently investigated by the excision of a region where BPC was sutured to the native porcine cornea, followed by tissue imaging by SEM.

### Subcutaneous implantation

Three Wistar rats weighing 150–200 g were given analgesic (0.01 mg Buprenorfin) by intraperitoneal injection the day before and on days 0, 1 and 2 post-surgery to minimize post-operative discomfort. While under general anesthesia (ketamine 25 mg/ml, Pfizer and dexmedetomidine hydrochloride 0.5 mg/ml, Orion Pharma), rat skin was shaved on the dorsal flank region, and a 2 cm-long para-vertebral cutaneous incision was made. A subcutaneous pocket was then created by blunt dissection, and a 1 cm square flat hydrogel was placed into the pocket. The pocket was then sealed with three Vicryl absorbable sutures. Images were taken on days 0, 3, 5, 7, 6 and 21, and 8 weeks post-surgery to evaluate wound healing and signs of inflammation. Rats were euthanized at 8 weeks, and tissue at the implantation site was collected for evaluation of extent of degradation, tissue fibrosis, leukocyte invasion and neovascularization. Rats were used after obtaining ethical approval from the Linköping Animal Ethical Review Board (Permit ID 585), and all experiments were in compliance with EU Directive 2010/63/EU.

### Animals and surgical models for femtosecond laser-assisted keratoplasty

After obtaining approval from the Linköping Animal Research Ethics Committee (Permit no. 108-12), 23 male New Zealand White albino rabbits weighing 3–3.5 kg underwent corneal operation. Experiments were conducted according to the Association for Research in Vision and Ophthalmology (ARVO) Statement for the Use of Animals in Ophthalmic and Vision Research. Animals were anesthetized by intramuscular injection of 25 mg/kg ketamine (Ketalar 50 mg/ml; Parke-Davis, Taby, Sweden) and 5 mg/kg xylazine (Rompun 20 mg/ml; Bayer, Gothenburg, Sweden). In addition to general anesthesia, local anesthetic drops (tetracaine hydrochloride eye drops 1%, Chauvin Pharmaceuticals Ltd., Surrey, UK) were also administered before surgery.

Rabbits were divided into four groups, distinguished by implant type (either native rabbit corneal tissue, termed ‘autograft’, or BPC implants) and by surgical implantation method (ALK or hybrid intrastromal LASIK flap combined with ALK performed under the flap, termed ‘Flap-ALK’). Three groups of 5 rabbits each underwent ALK-autograft, ALK-BPC, and Flap-ALK autograft procedures, while the remaining group of 8 rabbits underwent Flap-ALK BPC surgery. All ALK-BPC procedures were completed with 440 µm thick BPC, whereas Flap-ALK BPC procedures were intra-stromal and used 280 µm thick BPC. BPC thickness was thicker than the removed tissue, to compensate for a known thickness reduction of the material post-implantation due to de-swelling^12^.

An Intralase iFS 150 kHz femtosecond laser (Abbott Medical Optics, CA, USA) was used to perform all surgeries. The right eye of all animals underwent surgery while the left eye remained untouched. The thinnest average corneal thickness of 370 µm (determined by optical coherence tomography) was used for both femtosecond laser-assisted operation procedures. In femtosecond laser-assisted ALK, a central stromal button of native cornea with 7 mm diameter and 280 µm depth (including epithelium) was cut by the laser. The underlying posterior stroma and endothelium of 90 µm average thickness was left untouched. The native corneal button was manually removed and was either positioned back in its former position (autograft) or replaced with a BPC implant of 440 µm thickness and 7 mm diameter. Autografts were sutured to the surrounding host tissue with six to eight interrupted 10-0 nylon sutures, while due to the risk of a high suture tension tearing the biomaterial, BPC implants were held in place with three overlying 10-0 mattress sutures, as done previously^9^. In the femtosecond laser-assisted hybrid Flap-ALK procedure, the laser was first used to create a superficial corneal LASIK-type flap of 8 mm diameter and 100 µm thickness. Thereafter, underneath the flap a corneal stromal button of 7 mm diameter and 180 µm thickness was cut. The posterior corneal stroma and endothelium (around 90 µm thick) under the cut region were left untouched. After completing the laser cuts, the flap was manually separated and lifted with a blunt surgical spatula, and the underlying corneal button was removed using surgical forceps. In the autograft group, this corneal button was subsequently placed back in the stromal bed while in the BPC group it was replaced with a BPC implant of 280 µm thickness and 7 mm diameter. Following native stromal button or implant replacement, the overlying flap was closed over the stromal region and was secured to surrounding tissue with three to seven interrupted 10-0 sutures placed through the flap and peripheral host tissue (avoiding suturing the biomaterial or autograft button underlying the flap).

Immediately following surgery, a combination of hydrocortisone and oxytetracycline ointment (Terracortil with Polymyxin B 5 ml; Orifarm AB, Stockholm, Sweden) was topically administered and corneas were covered with a sodium hyaluronate viscoelastic (Healon GV 14 mg/ml; AMO Uppsala AB, Uppsala, Sweden) to protect exposed grafts in ALK procedures and to enhance epithelial healing in all operated corneas. Long-acting pain relief (Temgesic 0.3 mg/ml, RB Pharmaceuticals Limited, Berkshire, UK) was administered postoperatively by intramuscular injection to all rabbits every twelve hours during the first two postoperative days.

The steroid-antibiotic drops were administered three times daily the first postoperative week and were continued or reintroduced depending on the clinical evaluation of the operated eyes. In cases of severe pain that affected the general status of the rabbits, pain relief was also reintroduced. The sutures were removed one month after operation in all rabbits.

### Postoperative clinical assessment

Operated eyes were examined clinically by high magnification digital photography (Nikon D90 camera, Nikon Canada Inc., Toronto, Canada), anterior segment optical coherence tomography (AS-OCT; Visante OCT, Carl Zeiss AB, Stockholm, Sweden) and in vivo confocal microscopy (IVCM; Heidelberg Retinal Tomograph 3 with Rostock Corneal Module, Heidelberg Engineering, Heidelberg, Germany). Examinations were conducted directly after operation and postoperatively after 1 week and 1, 3 and 6 months. Additionally, eyes were clinically evaluated for corneal opacity and neovascularization using the Modified MacDonald-Shadduck Scoring System^16^. Epithelial integrity of the operated corneas was examined one week after the operation with the fluorescein eye stain test.

As previously described^12,13^, AS-OCT was used to evaluate corneal thickness following surgery as well as to visualize the flap and the underlying implanted materials. The average central corneal thickness was determined automatically by the built-in OCT software. IVCM was also conducted in order to evaluate the morphology of the implants and all layers of the surrounding host corneal tissue, as per an earlier protocol^17^. Attention was also given to implant-to-host interfaces to detect possible host cell and nerve ingrowth into the implanted regions. IVCM images acquired 6 months postoperatively in all animals were used for qualitative and semi-quantitative analyses.

### Immunohistochemistry

After completion of corneal implantation in rabbits (6 months), corneas were excised and fixed in 4% paraformaldehyde solution, embedded in paraffin, sectioned to 4 µm- thickness and stained with hematoxylin and eosin (H&E). Sections from paraffin-embedded tissues were de-paraffinized with descending concentrations of xylene and ethanol, trypsinized for 5 min to retrieve antigen and endogenous peroxidase was neutralized with 3% hydrogen peroxide in methanol. Following blocking with bovine serum albumin (BSA) 2.5% for 1 h, sections were incubated overnight at 4 °C with the following primary antibodies: mouse monoclonal α-SMA (dilution 1:25, ab7817, Abcam, Cambridge, United Kingdom), mouse monoclonal beta III tubulin (dilution 1:100 , ab7751, Abcam), mouse monoclonal type III collagen (dilution 1:100, Acris AF 5810, Germany), and mouse monoclonal CD45 (dilution 1:10, ab86080, Abcam) in 2.5% BSA. Control sections were incubated with 2.5% BSA alone without the addition of the primary antibody. Sections were washed in PBS-T and incubated with HRP-conjugated IgG antibody (AP308P, 2688593; 1:1000; Merck Millipore, MA, USA) for 1 h at RT. Bound peroxidase was visualized by incubating the sections for 6 min in a solution of 0.1 M Tris–HCl (TB; pH 7.4) containing 0.05% 3-3′-diaminobenzidine (DAB, Sigma-Aldrich), 0.04% nickel chloride and 0.0075% H_2_O_2_. The reaction was stopped by several washes with PBS-T. Samples were then dehydrated, cleared in xylene and a coverslip was placed using Mountex mounting medium (Histolab Products AB, Gothenburg, Sweden). Light microscopy was performed with an Axiophot Photomicroscope (Zeiss, Germany) under 10× and 20× magnification.

Upon completion of subcutaneous implantation in rats (8 weeks), implants with surrounding soft tissues were removed and processed as described above. The same antibodies were used, but were visualized using the DyLight 488 secondary antibody (1:1000, Thermo Fischer Scientific, MA, US), and mounted (ProLong Gold antifade reagent with DAPI, Invitrogen, Thermo Fisher Scientific, MA, US). Images were taken with an LSM700 upright laser confocal microscope (Carl Zeiss, Oberkochen, Germany).

### In vitro drug release test

To evaluate the possibility of the BPC serving as a reservoir promoting sustained release drug delivery after implantation, an in vitro drug release test was performed. Recombinant rat nerve growth factor beta (NGF-β) was purchased (Sigma Aldrich, Stockholm, Sweden) and a stock solution of 3.2% w/v concentration was prepared by dissolving NGF-β powder in sterile MES buffer solution (2-(N-morpholino)ethanesulfonic acid). The solution was maintained at room temperature and 48 µl was incorporated into collagen stock solution during manufacture of the BPC implants. During implant manufacture and storage, samples from the supernatant of the storage medium were saved and used to determine the total amount of NGF-β in the supernatant, which was subtracted from the initial amount added during manufacturing, to determine the total mass of NGF-β in each BPC implant. For the slow release test, six implants (experimental replicates) were incubated separately in 2 ml of sterile PBS and maintained at 37 °C for a period of 60 days. Sampling time points were as follows: 6 h, 24 h, 3d, 5d, 10d, 20d, 30d, 40d, 50d, and 60d. At each sampling time point, 200 µl of supernatant was withdrawn and stored at − 20 °C and replaced with 200 µl of fresh sterile PBS. After 60 days, all samples were thawed and analyzed by ELISA (Rat NGF-β ELISA Kit, Sigma) according to the manufacturer instructions. Serial dilution of the NGF-β stock solution was used to establish a standard calibration curve. The cumulative release profile was determined at each time point as the sum of total NGF-β in the total supernatant volume (concentration × total volume) at the present and all earlier time points. This cumulative released amount was then expressed as a percentage of the total mass of NGF-β initially present within each BPC implant.

### Statistical analysis

Corneal thickness at 6 months in autograft, BPC implanted, and native rabbit corneas was compared by one-way ANOVA applied to ALK and Flap-ALK methods, with Tukey’s post-hoc comparison method to isolate pairwise differences. Corneal opacity and neovascularization score in autograft and BPC implanted corneas at 6 months were compared by the Mann–Whitney rank sum test. All statistical analyses were performed using SigmaStat version 3.5 for Windows (Systat Software Inc., Chicago, USA). A two-tailed threshold of *P* < 0.05 was considered statistically significant.

## Results

### Physical properties of BPC implants

**Figure 1:**
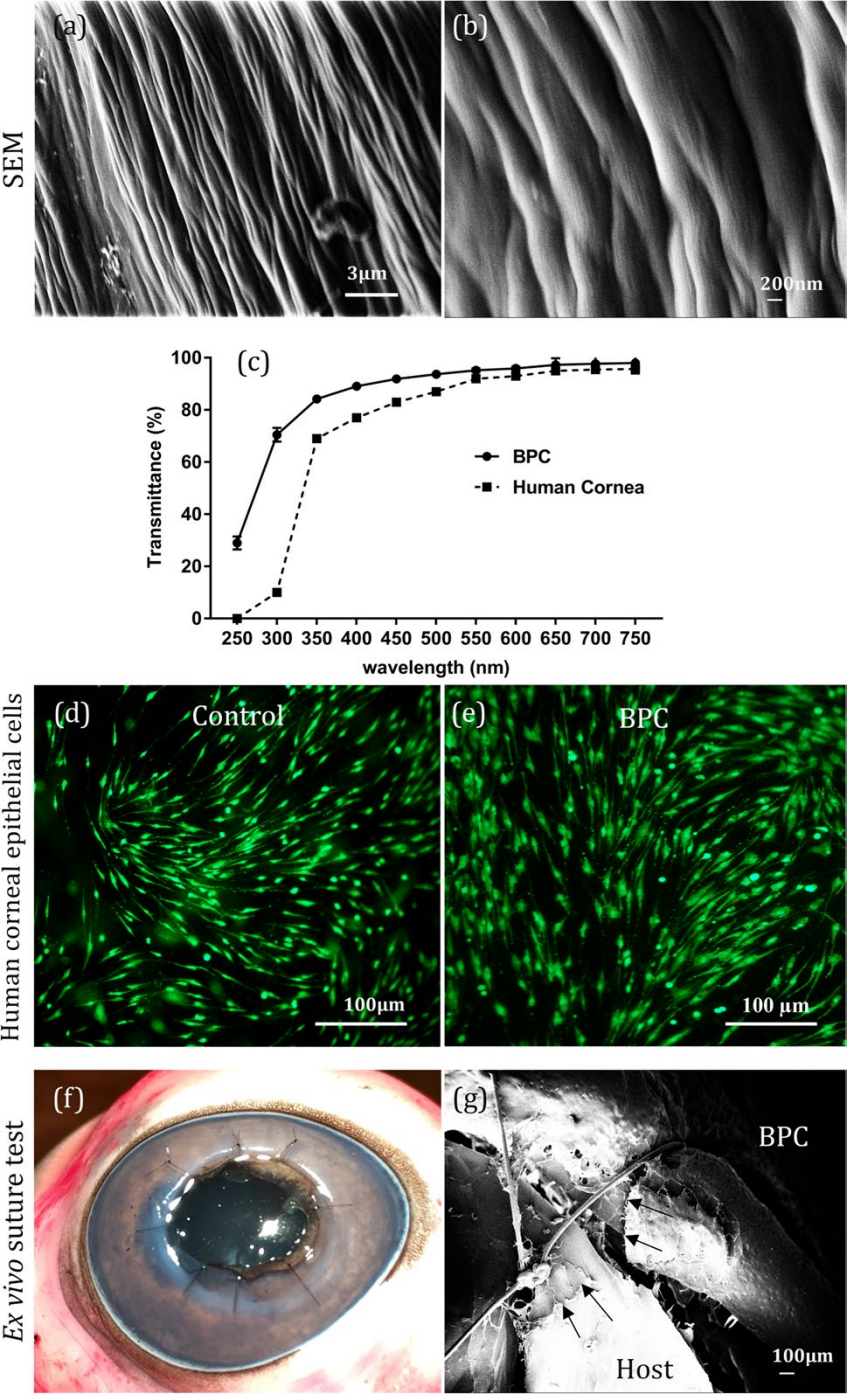
Properties of the bioengineered porcine construct (BPC). Scanning electron microscope images of the BPC surface morphology indicating long, regularly arranged parallel-running collagen fibers at low (**a**) and high (**b**) magnifications. (**c**) Optical transmittance curve of the BPC hydrogel relative to the human cornea^15^. (**d**) Human corneal epithelial cells cultured in vitro without BPC and (**e**) on top of the BPC hydrogel. Green fluorescence indicates live cells. (**f**) Test of direct suturing of BPC into a porcine eye ex vivo. The BPC was sutured into the surrounding pig cornea with 8 interrupted sutures. (**g**) SEM images at the interface of the sutured hydrogel with the host cornea, indicating microfractures created by the suture cutting through the BPC and host cornea (arrows).

BPC hydrogels demonstrated a macroscopically smooth implant surface with fine structure visible by SEM (Fig. 1). At the nanoscale, the implant surface was characterized by collagen fibers arranged in a parallel, unidirectional configuration (Fig. 1a, b). Optical transmission in vitro was equivalent or superior to the human donor cornea stored in standard DMEM for each tested wavelength, with average BPC transmission of about 90% in the visible wavelength range (Fig. 1c). We have previously reported the mechanical properties of the BPC material relative to the healthy human donor cornea^12^.

### In vitro cell compatibility of BPC

In vitro, the proliferation of viable HCEC was observed when seeded onto BPC for 14 days in culture. HCEC viability on BPC (from two different human donors) resembled that of medium-only positive controls where cells were grown on culture plates with only culture media added, without the presence of BPC (Fig. 1d, e). The metabolic activity as a measure of cell viability and their growth on the implants was evaluated using the MTT assay. Cells seeded onto BPC had a cell viability of 83.46 ± 15.32% (donor sample 16,188) and 87.33 ± 2.65% (donor sample 16,200), relative to positive controls.

### Suturability of BPC implants

An ex vivo procedure was used to implant BPC into whole porcine eyes by penetrating keratoplasty. All BPC implants of 440 µm thickness could be sutured into recipient porcine corneas, with most of the knots buried into the surrounding recipient stroma without macroscopic cracking of the BPC material (Fig. 1f). Microscale imaging of the recipient-implant interface region with the suture by SEM revealed, however, micro-fractures in the biomaterial induced by the suture material under high tension (Fig. 1g). Because of a presumed increased risk of tearing the BPC when sutured in vivo into eyes with a dynamic environment (physiologic intraocular pressure and constant eyelid motion), an overlying mattress-type suture pattern was instead applied to secure the BPC implants within the recipient cornea in the ALK procedure^9^. The Flap-ALK procedure, however, does not require direct suturing of the implant and thus avoids direct suture contact.

### Evaluation of subcutaneous implantation of BPC in rats

**Figure 2:**
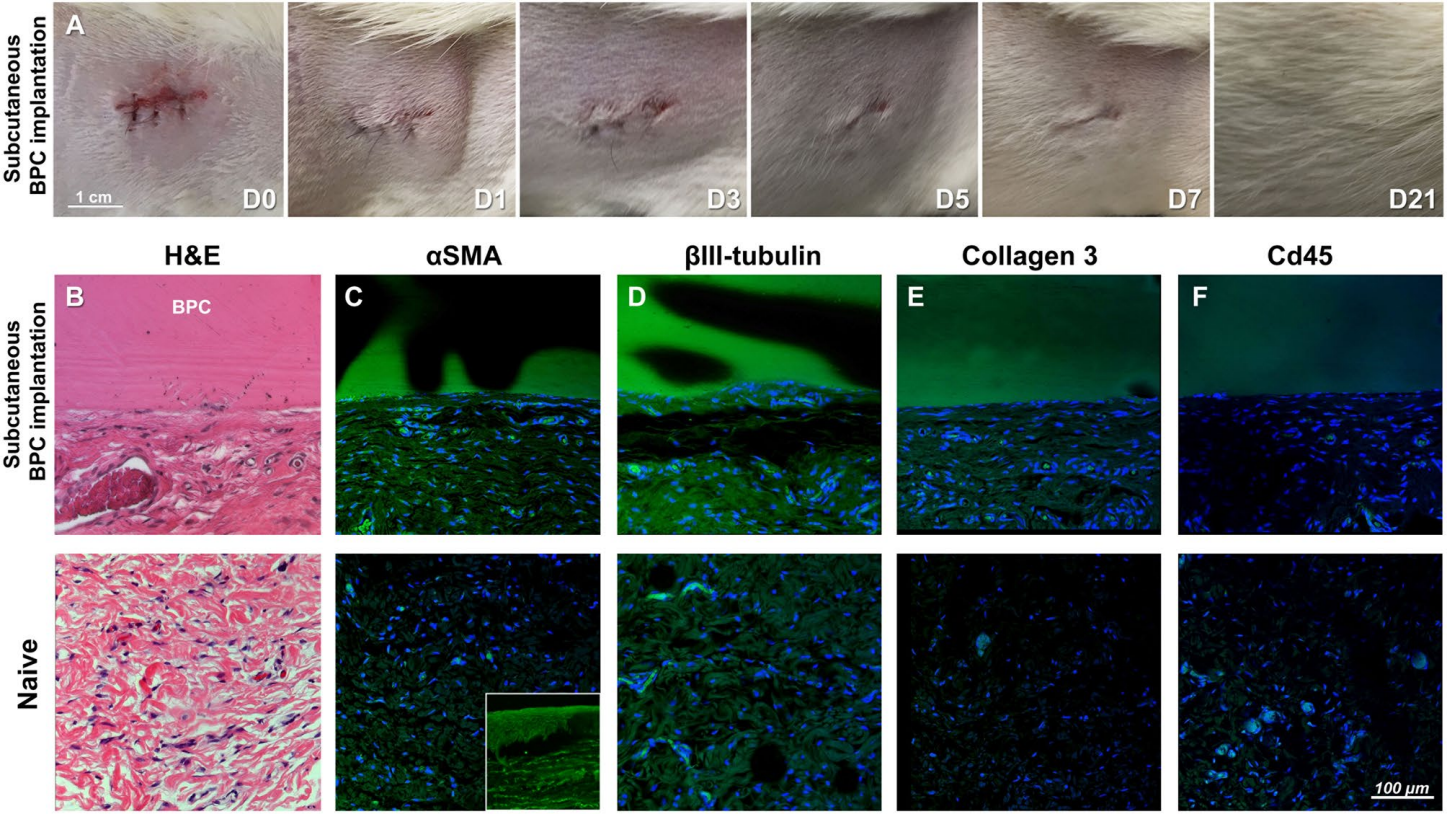
Subcutaneous implantation of the BPC hydrogel in rats. (**a**) Serial images of the same implanted dorsal flank region from day 0 to 21, indicating wound healing without overt inflammation. (**b**) Hematoxylin and eosin stained sections of the implanted and control region of tissue indicate apposition of the BPC to native tissue without degradation after 8 weeks. (**c**–**f**) Immunohistochemical staining indicates some activation of αSMA, type III collagen, and βIII-tubulin in the interface region. No CD45 positive leukocytes were found in the tissue adjacent to the BPC implant. A positive control of an inflamed cornea with high activation of myofibroblasts and high positivity for αSMA staining can been seen in the smaller inset figure in (**c**).

Prior to in vivo implantation in the eye, the biocompatibility of BPC hydrogels was evaluated by subcutaneous implantation in rats for 8 weeks. Following implantation, healing of the surgical wounds occurred without complications, with no signs of infection or acute inflammation such as redness, discoloration or itching. The wounds had fully healed during a period of 1–3 weeks post-implantation (Fig. 2a). After 8 weeks, BPC implants were retrieved for immunohistochemical analysis, where hematoxylin and eosin staining revealed intact BPC implants with minimal cellular infiltration at the interface with host tissue (Fig. 2b). Implantation induced mild myofibroblast activation indicated by α-SMA staining (Fig. 2c) while partial expression of neuronal marker β-III tubulin was reestablished near the implant interface by 8 weeks (Fig. 2d). The implant-to-host interface region was also characterized by type III collagen deposition (Fig. 2e). An absence of CD45+ leukocytes indicated the implanted site was quiescent and devoid of persistent inflammatory reaction (Fig. 2f).

### Surgeries and surgical complications in rabbit corneal implantation models

**Figure 3:**
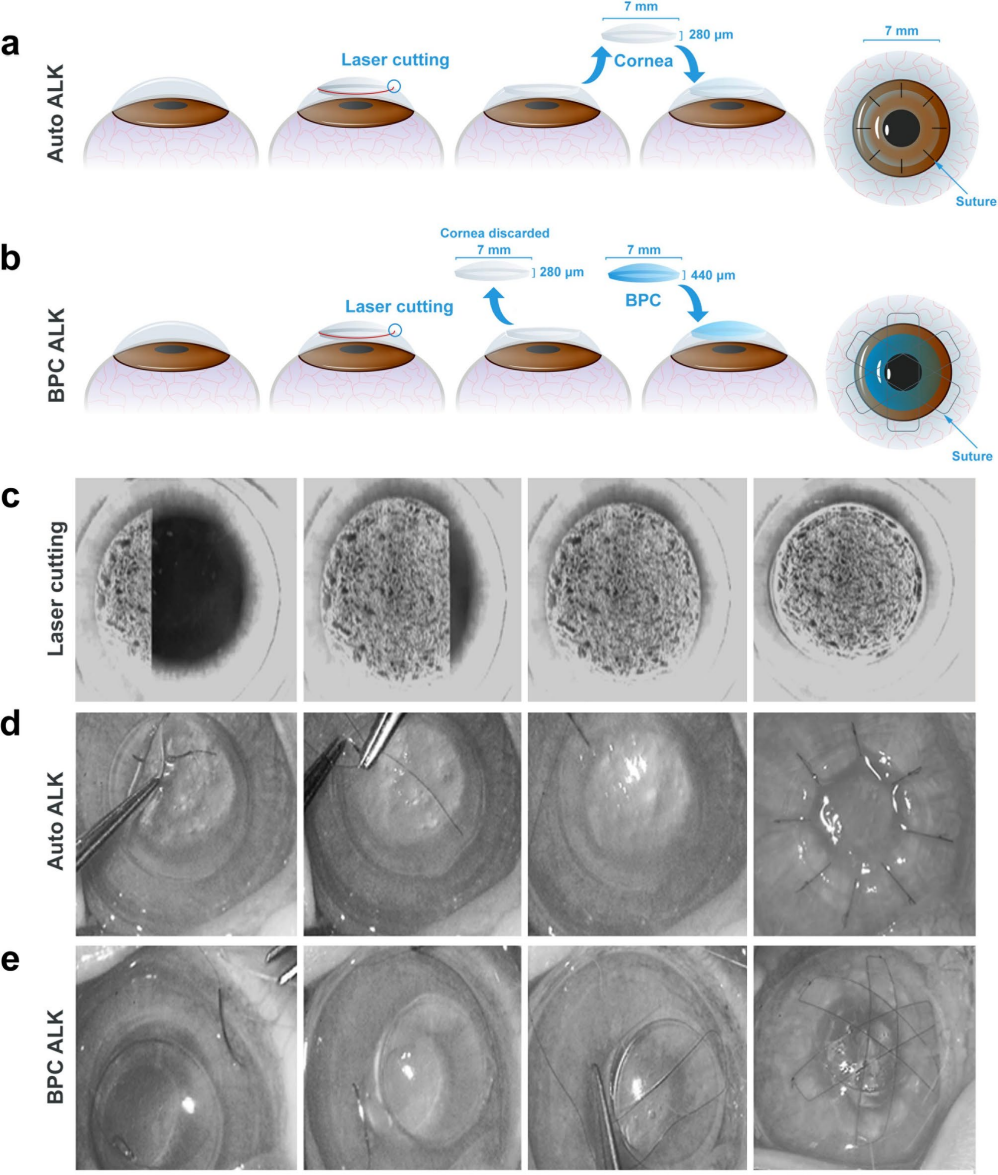
Surgical technique for femtosecond laser-assisted anterior lamellar keratoplasty (ALK). (**a**) Procedure for autograft tissue. A femtosecond laser cuts a disc of stromal tissue, which is then replaced in the same bed and sutured with eight interrupted sutures. (**b**) For BPC hydrogel implants, the disc of removed native tissue is instead discarded and replaced by the BPC hydrogel. Overlying mattress sutures are then placed to avoid suturing through the hydrogel. (**c**) Time-lapse images of the laser cutting the disc of stromal tissue. (**d**) Time-lapse photographs of the surgical implantation procedure, showing extraction of the stromal disc and replacement of the same disc or the BPC hydrogel, followed by placing interrupted (autograft) or (**e**) mattress-type (BPC) sutures.

**Figure 4:**
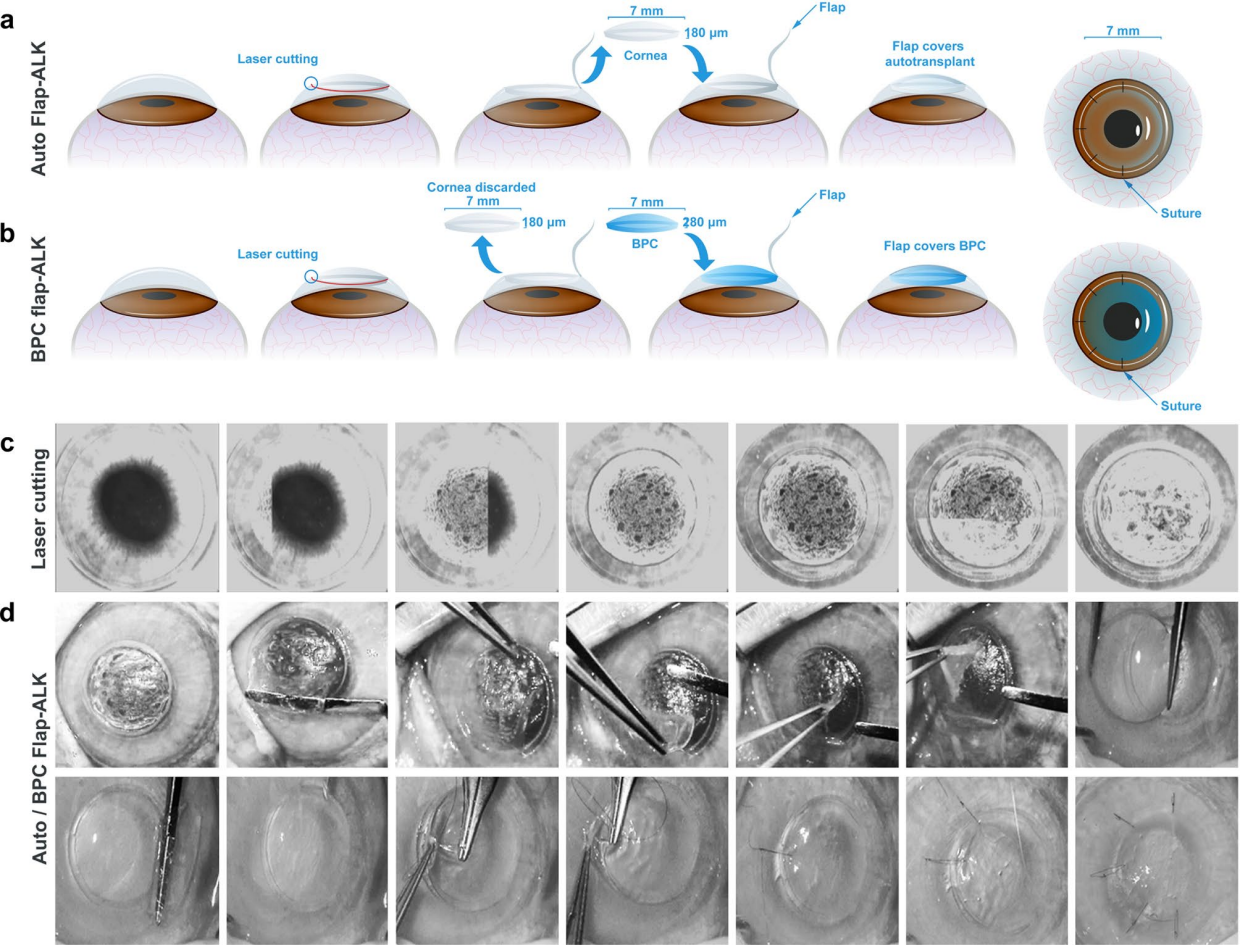
Surgical technique for the hybrid Flap-ALK procedure. (**a**) Procedure for autograft tissue. A femtosecond laser cuts the flap and underlying lamellar tissue in a single session. The flap is lifted, the underlying lamellar stromal disc is removed, then replaced in the same bed. The flap is then closed and sutured in place with five interrupted sutures. (**b**) For BPC hydrogel implants, the disc of removed native tissue is instead discarded and replaced by the BPC hydrogel. The flap is laid over the implant and sutured by the same technique as for allografts. (**c**) Time-lapse images of the laser cutting. First the deeper lamellar button is cut, followed by the larger diameter flap. (**d**) Time-lapse photographs of the surgical implantation procedure, showing flap opening, extraction/peeling of the stromal button, and replacement with the BPC hydrogel, followed by flap coverage and suturing the flap.

During corneal surgery in rabbits, several intraoperative surgical complications occurred, including a flap tear during flap separation from the underlying stroma (1 case), and small localized puncture of endothelium during ALK, prior to material implantation (3 cases). Apart from these complications, surgeries were completed successfully with BPC materials being held in place by overlying mattress sutures and autografts by interrupted sutures in ALK (Fig. 3). In the hybrid Flap-ALK procedures, all implants (BPC and autograft) underwent the same suturing technique, with sutures drawn through the native flap tissue only, and secured to peripheral native corneal tissue (Fig. 4).

Several sutures had loosened or were lost one week postoperatively in all treatment groups. Steroids were applied the first postoperative week in all animals; however, 3 eyes required prolonged administration of topical steroid to a maximum of 3 additional weeks due to neovascularization and/or postoperative inflammation (1 autograft ALK, 1 autograft Flap-ALK, 1 BPC Flap-ALK). These eyes remained in the study. A severe inflammatory reaction with purulent excretion was detected in one eye and led to premature euthanasia two weeks postoperatively (BPC Flap-ALK). Additionally, one animal was lost due to general anesthesia-related stress following the 3-month postoperative examination (BPC Flap-ALK).

### Postoperative corneal thickness, transparency, and neovascularization

**Figure 5:**
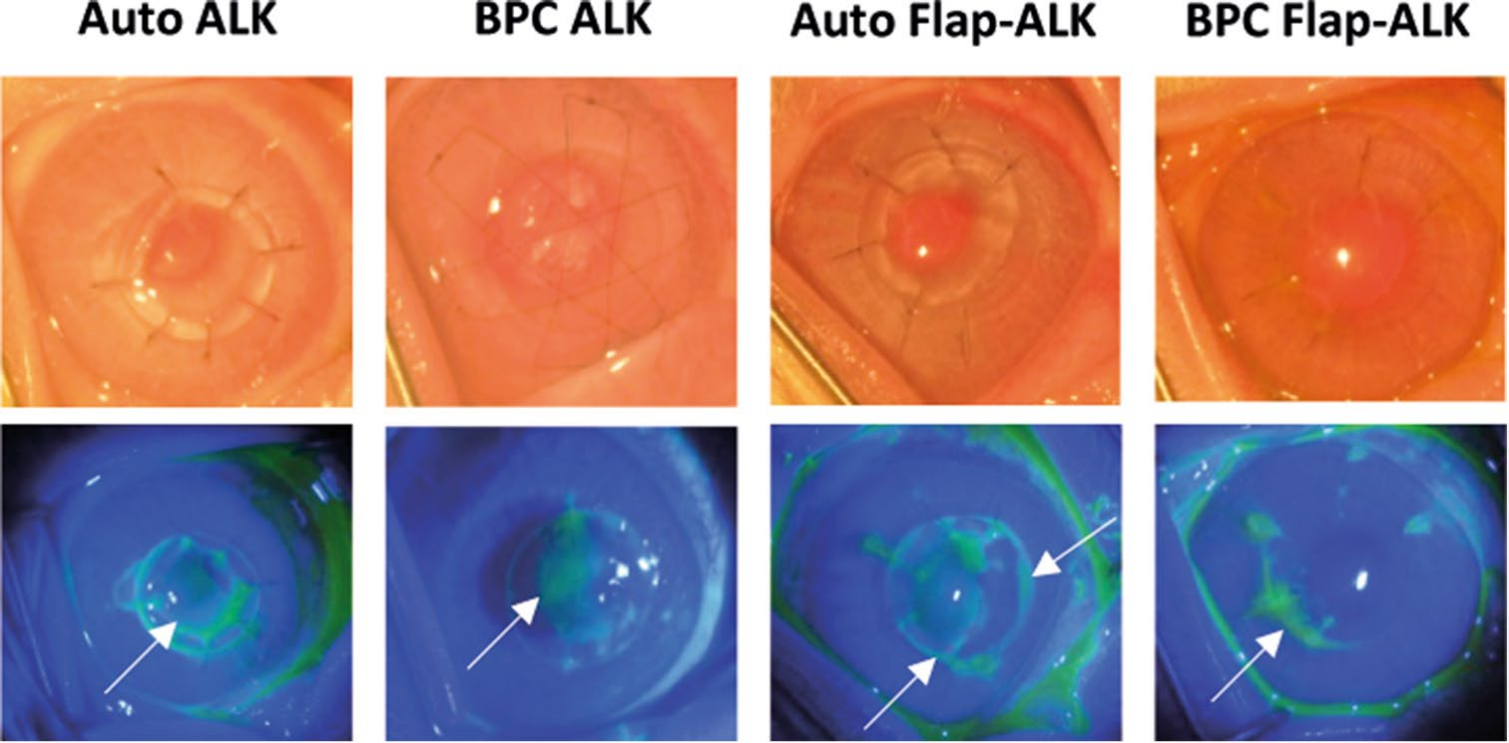
Fluorescein staining one week postoperatively. Incomplete epithelial coverage of implants in the ALK model was apparent (arrows indicate non-epithelialized areas in green fluorescence). Corneas in the Flap-ALK model retained the native epithelium except for a thin region at the flap border (arrows) with incomplete epithelial closure. By one month, all epithelial wounds had healed.

**Figure 6:**
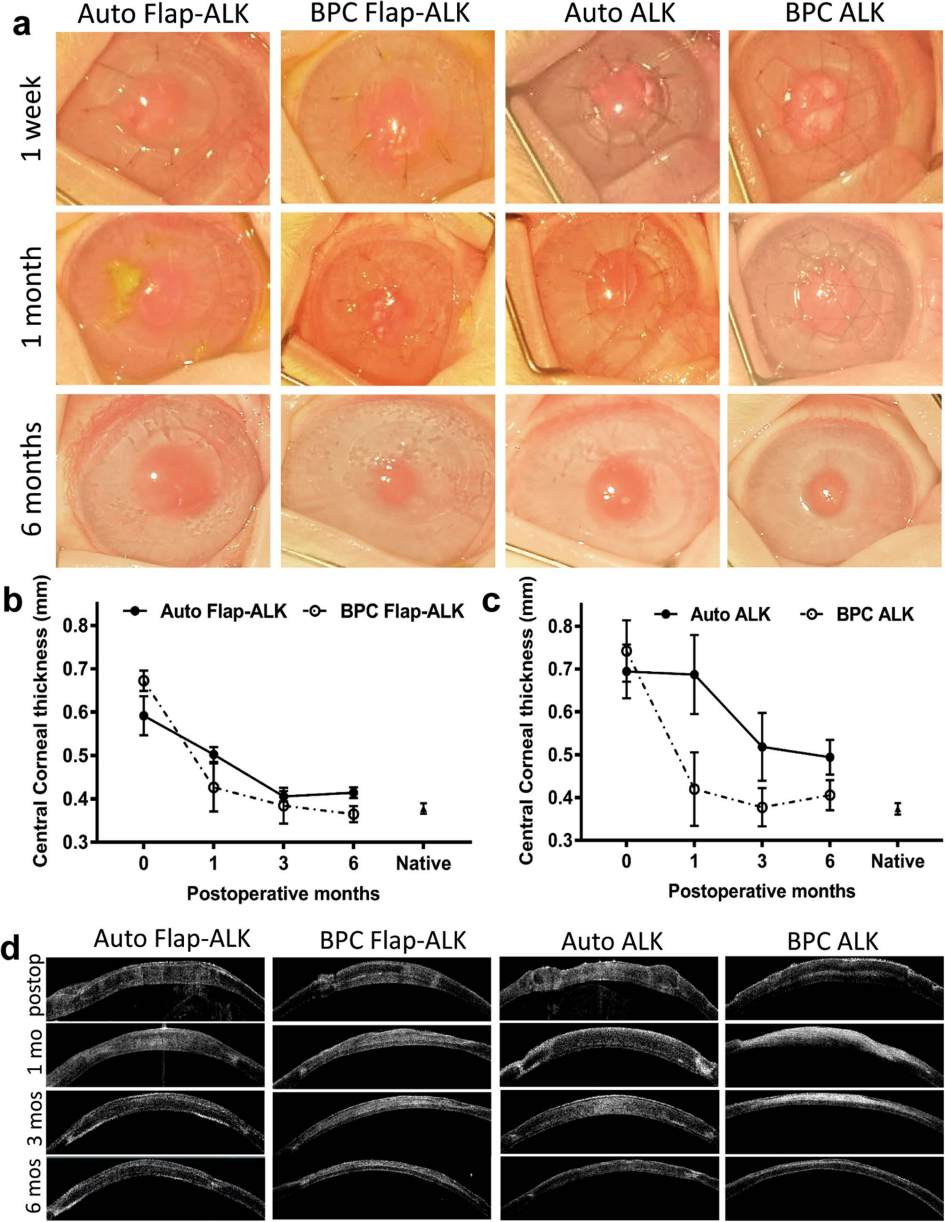
Postoperative corneal appearance, thickness and AS-OCT scans. (**a**) Longitudinal clinical in vivo photographs of the same corneas up to 6 months postoperatively. (**b**) Central corneal thickness in autograft and BPC groups with the Flap-ALK procedure. (**c**) Central corneal thickness in all groups based on AS-OCT measurements. (**d**) Longitudinal AS-OCT photographs of the same corneas postoperatively. BPC implants by the Flap-ALK procedure maintained corneal thickness and curvature postoperatively, while BPC implants by the ALK procedure had thinned. Error bars in (**b**) indicate standard error of the mean (SEM).

During the first postoperative week, a mild corneal haze (reduced transparency) was present in all groups (including autograft controls) and epithelial wound healing was assessed by clinical photography with fluorescein staining (Fig. 5). At one week, ALK grafts had not completely epithelialized, while in Flap-ALK procedures the central epithelium was intact but the flap border with host tissue was not completely covered by epithelium. After 1 month, sutures were removed and by 6 months the iris and pupil structures were visible (Fig. 6a). Epithelium was fully restored in all groups and no fluorescein staining was observed in any cornea (data not shown). The Flap-ALK method led to a final corneal thickness closer to the native rabbit cornea than the ALK method (Fig. 6b,c). Final corneal thickness at 6 months in the Flap-ALK method differed between materials (ANOVA P = 0.033) with autografts significantly thicker than BPC (*P* = 0.026), while BPC and native corneal thickness did not differ (*P* > 0.05). With the ALK method, thicknesses differed significantly at 6 months depending on material type (ANOVA *P* = 0.026), with autograft significantly thicker than the native rabbit cornea (*P* = 0.022) while BPC corneal thickness did not differ from native corneas (*P* > 0.05). Longitudinal AS-OCT scans revealed persistent postoperative edema in autograft-implanted corneas while BPC implants by 6 months either maintained a normal corneal thickness (Flap-ALK) or exhibited partial decrease in thickness (ALK) postoperatively (Fig. 6d). At 6 months postoperative, the anterior corneal curvature had normalized and followed the curvature of the posterior cornea in all implanted eyes.

**Figure 7:**
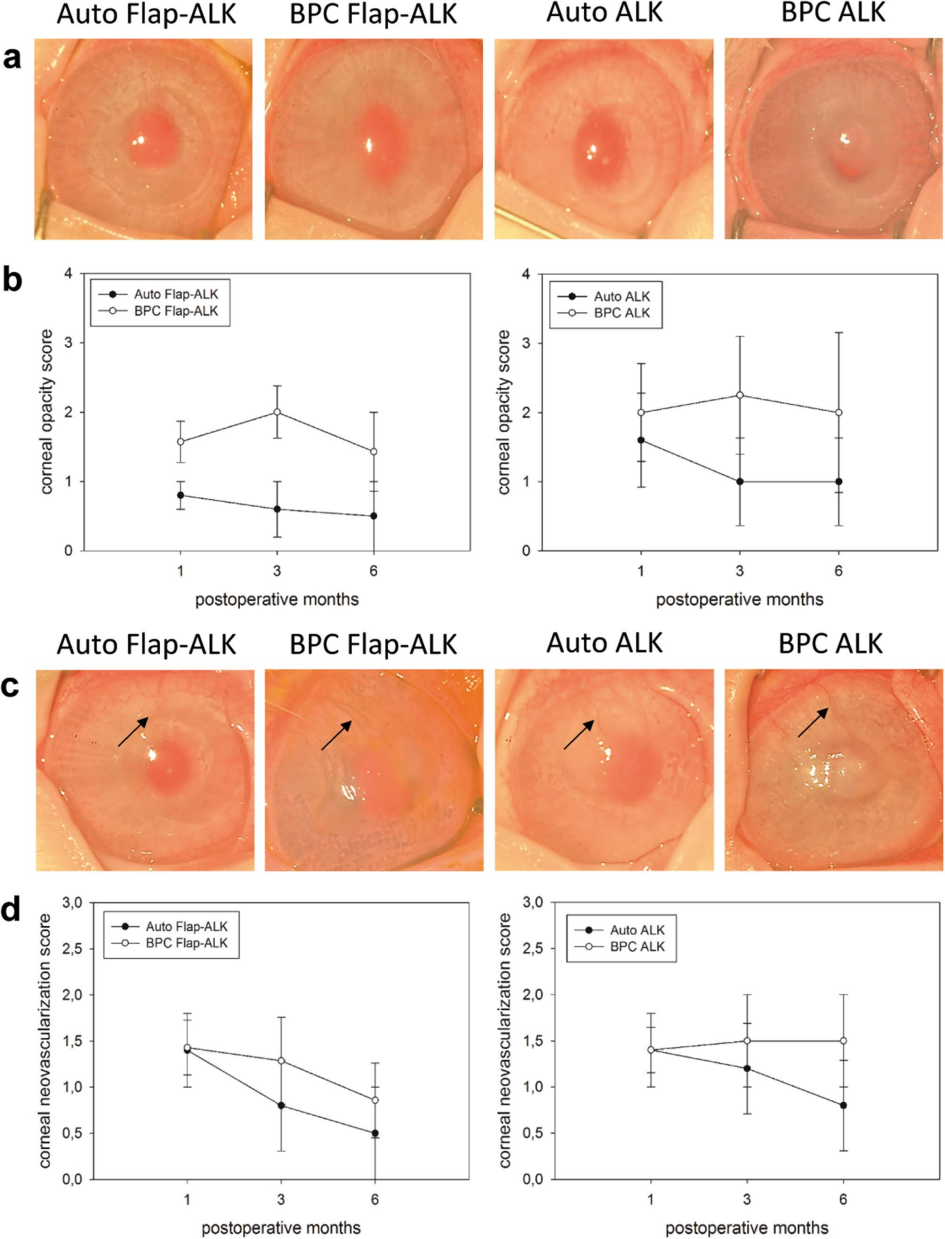
Clinical scoring of corneal opacity and neovascularization in implanted corneas. (**a**) Clinical appearance of corneas at 6 months. (**b**) Longitudinal opacity score for corneas implanted by both methods. (**c**) Clinical appearance at 6 months indicating new invading vessels (arrows) entering the host cornea and terminating at the implant periphery in all groups. (**d**) Longitudinal neovascularization score for eyes implanted by both methods. Error bars indicate SEM.

Corneal transparency scoring by clinical evaluation (Fig. 7a) indicated reduced opacity in autografts relative to BPC implanted corneas in the early postoperative period (Fig. 7b); however, at 6 months no difference was apparent between the implant types (*P* = 0.41 for Flap-ALK, *P* = 0.45 for ALK). Neovascularization was observed in all groups at 1 month with peripheral vessels invading the cornea in the location of the sutures in the peripheral implanted zone (Fig. 7c). Neovascularization scores for autograft and BPC implanted groups were similar at 1 and 6 months (Fig. 7d; Flap-ALK, *P* = 0.65; ALK, *P* = 0.36).

### In vivo assessment of corneal morphology

**Figure 8:**
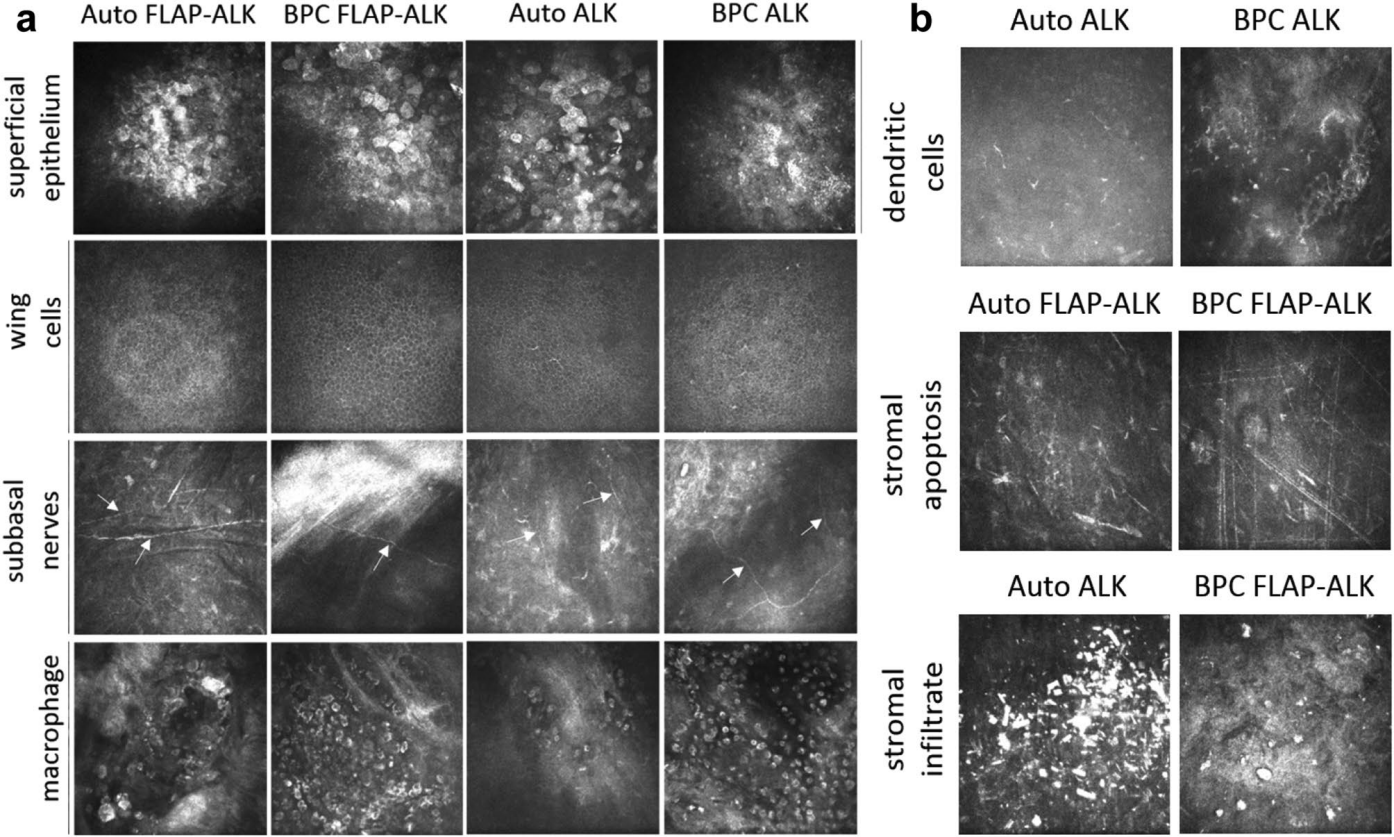
Morphology of implanted corneas in vivo 6 months postoperatively, as imaged by laser-scanning in vivo confocal microscopy (IVCM). (**a**) Superficial epithelial cells, epithelial wing cells, subbasal epithelial nerves (arrows) and infiltrating macrophages were present in all groups to varying degrees. (**b**) In vivo detection of inflammatory dendritic cell invasion into the central subepithelial cornea, apoptotic remnants in the stroma (indicated by short and long linear structures), and stromal infiltrates consisting of hyper-reflective deposits and debris in the flap interface (reflective rounded structures). All IVCM images are 400 × 400 µm.

IVCM was performed to examine the anatomic layers within the host corneal tissue and implants at the cellular level, including superficial epithelium, wing cells, subbasal nerves, macrophages, dendritic cells, stromal apoptosis and stromal infiltrate^18^. At 6 months, a stratified epithelium with superficial and wing cells was observed in all groups (Fig. 8a). Subbasal nerves were visible directly beneath the epithelium of the central cornea in all groups, indicating partial regeneration of the subbasal nerve plexus. Macrophages were additionally detected in the stromal implant region in all groups (Fig. 8a). Dendritic cells in the epithelium were only detected in two corneas (autograft ALK and autograft Flap-ALK, Fig. 8b). Small particulate bodies and linear structures indicative of stromal cell apoptosis^19^ were found in all groups except the BPC ALK group (Fig. 8b). In the Flap-ALK procedure, apoptosis was restricted to the implant-flap interface. Round, hyper-reflective structures were found in a minority of corneas in all groups except the BPC ALK group (Fig. 8b). These formations were interpreted as a stromal infiltrate due to surgical debris or degradation products from infiltrating macrophages. The frequency of detection of the various morphologic features in implanted eyes is summarized in Table 1.

**Table 1:** Frequency of in vivo microscopic findings in the cornea in the various treatment groups.

	Superficial cells	Wing cells	Subbasal nerves	Dendritic cells	Stromal apoptosis	Stromal infiltrate	Macrophage infiltration
Autograft ALK (5)	4/5	5/5	1/5	1/5	2/5	1/5	1/5
BPC ALK (4)	3/4	2/4	2/4	1/4	0/4	0/4	1/4
Autograft Flap-ALK (4)	3/4	2/4	2/4	0/4	3/4*	1/4	1/4
BPC Flap-ALK (5)	5/5	5/5	2/5	0/5	2/5*	1/5	2/5

The number of animals per group with images of sufficient quality for examination is given in parentheses.

An asterisk * indicates that apoptosis was limited to the flap interface region.

### Histopathologic evaluation of implanted corneas

**Figure 9:**
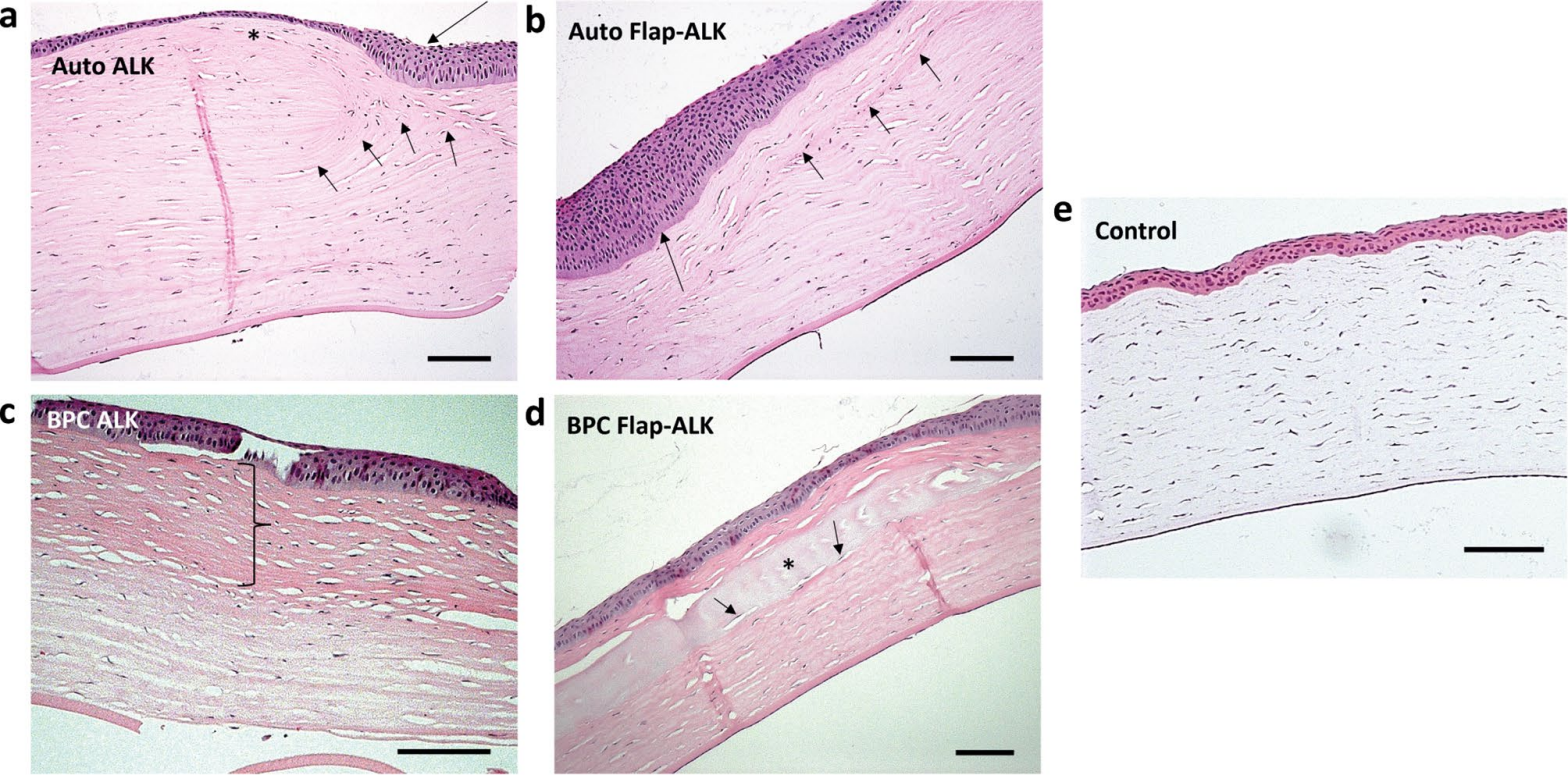
Hematoxylin and eosin staining of representative sections from untouched controls, autograft and BPC implanted corneas 6 months postoperatively. (**a**) In autografts implanted by ALK, tissue and cells at the implant edge were deformed in a manner suggesting mechanical tension (short arrows). At the edge of the implant region, a thickened epithelium was apparent (long arrow), while a thin epithelium was present above the implant (asterisk). (**b**) In the Flap-ALK model, autograft tissue was indistinguishable from the native stroma, apart from a line of denser stromal collagen (short arrows) indicating the posterior flap border. The epithelium was thickened at the edge of the flap (long arrow). (**c**) For BPC implanted by ALK, the BPC implant was transformed into a layer of denser, strongly eosinophilic collagen (demarcated zone) within which host stromal cells were present. (**d**) By contrast for BPC implanted by the Flap-ALK method, the BPC hydrogel was readily identifiable by strong hematoxylin staining (asterisk), with host stromal cells also visible within the material (arrows). (**e**) The multilayered epithelium of the untouched cornea has the same thickness through the whole surface and the stroma is composed of collagen lamellae with quiescent keratocytes. Scale bars: 100 µm.

Histological examination of the cornea at 6 months postoperative was performed with hematoxylin and eosin stained corneal sections (Fig. 9). In the autograft ALK group the corneal stroma was thick, and additionally had a thickened epithelium present at the edge of the implanted region. The native corneal tissue was hypothesized to have been under mechanical tension due to linear patterns in the stromal collagen and distribution of stromal cells oriented towards the suture location at the edge of the implanted region. The BPC ALK group, by contrast, had thickness resembling the native rabbit cornea; however, the implanted BPC material was not clearly identifiable as a cell-free implant but appeared to be partially or wholly replaced by dense, eosin-positive stromal tissue containing cells in all cases. With the Flap-ALK method, in autografts the posterior interface of the flap was identifiable as a line of denser stromal collagen, while the underlying native tissue was indistinguishable from the native stroma. The flap closure site, however, was characterized by thickened, stratified epithelium. For BPC-implanted corneas in the Flap-ALK method, the implanted material was clearly identified by blue-purple hematoxylin positivity, and the corneal stroma and epithelium had a thickness and stratification resembling the native rabbit cornea. The flap region anterior to the implant consisted of a denser, more compacted collagen compared to the native posterior stroma. The BPC was adherent to the surrounding stroma without gross stromal or cellular distortions.

**Figure 10:**
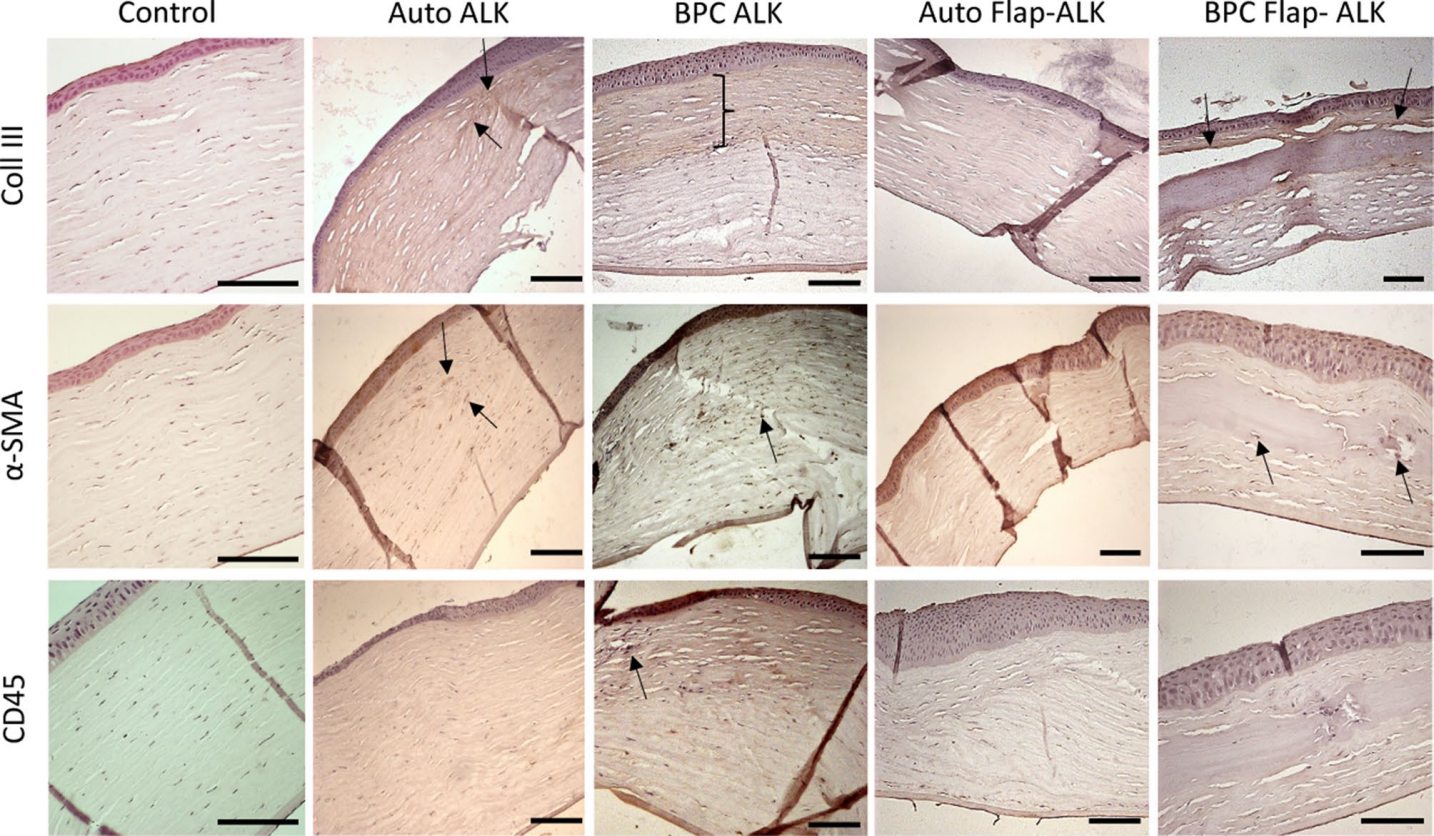
Immunohistochemistry analysis of operated rabbit corneas 6 months postoperatively in the various treated groups compared to untouched control cornea. In the autograft ALK group, minimal type III collagen was expressed, limited to the edge of the implant (arrows), where α-SMA positive fibroblasts (arrows) were also present. In the ALK group receiving BPC implants, stronger type III collagen expression was apparent in the anterior cornea (marked zone), with this implanted zone no longer containing a cell-free implant. Instead, α-SMA positive fibroblasts (arrow) were present in the anterior stroma, along with isolated CD45-positive leukocytes (arrow). In the autograft group subjected to the Flap-ALK implantation method, no fibroblasts or leukocytes were apparent, nor was type III collagen expressed. Where BPC was implanted under the flap, a thin region of type III collagen tissue was apparent (arrows) at the interface. Additionally, α-SMA positive fibroblasts (arrows) were present within the BPC, but no leukocytes were detected in the stroma. The untouched cornea, on the contrary, had negative staining for type III collagen and cells were negative for α-SMA, with only a few CD45 positive cells present. Scale bars: 100 μm.

**Figure 11:**
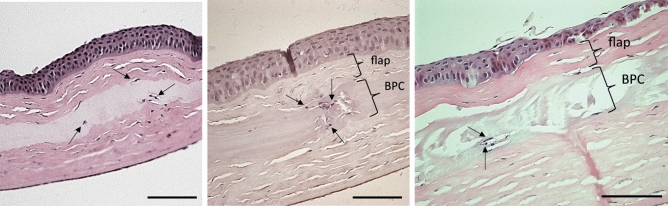
Host stromal cell migration into BPC implants in several rabbit corneas 6 months postoperatively, where the Flap-ALK implantation method was used. All corneas in this group exhibited stromal cell migration into the implant region. The arrows indicate host cells within the BPC implant. Scale bars: 100 μm.

Immunohistochemical staining (Fig. 10) revealed only a mild, diffuse expression of the fibrotic type III collagen in autografts implanted by ALK. Stronger expression of type III collagen was confined to the edges of the implanted region, which additionally contained stromal cells expressing α-SMA. No CD45+ cells were detected in the stroma 6 months postoperatively. In corneas with BPC implanted by the ALK method, the entire anterior implanted zone of the corneal stroma under the epithelium stained positive for type III collagen, while the cells within this zone expressed α-SMA, with a few cells in this region expressing CD45. In the Flap-ALK method, autografts did not express collagen III, and stromal cells did not express α-SMA. Although the stroma and epithelium appeared thickened and distorted, particularly near the edge of the implant zone, no CD45+ cells were detected in the stroma. For BPC implanted by the Flap-ALK method, a thin layer staining positive for collagen III was apparent at the anterior interface of the BPC implant with the posterior flap. Stromal cells observed at the BPC-stromal interface and within the BPC expressed α-SMA but not CD45. The migration of native stromal cells into the BPC implant by 6 months postoperatively was not an isolated phenomenon but was observed in all BPC-implanted corneas in the Flap-ALK model (Fig. 11). In cases of stromal cell population of the BPC, minimal degradation of BPC occurred in the immediate proximity of the infiltrating cells, as indicated by regions of absent extracellular matrix adjacent to these stromal cells (Figs. 9, 10, 11).

### In vitro release of NGF-β from BPC materials

**Figure 12:**
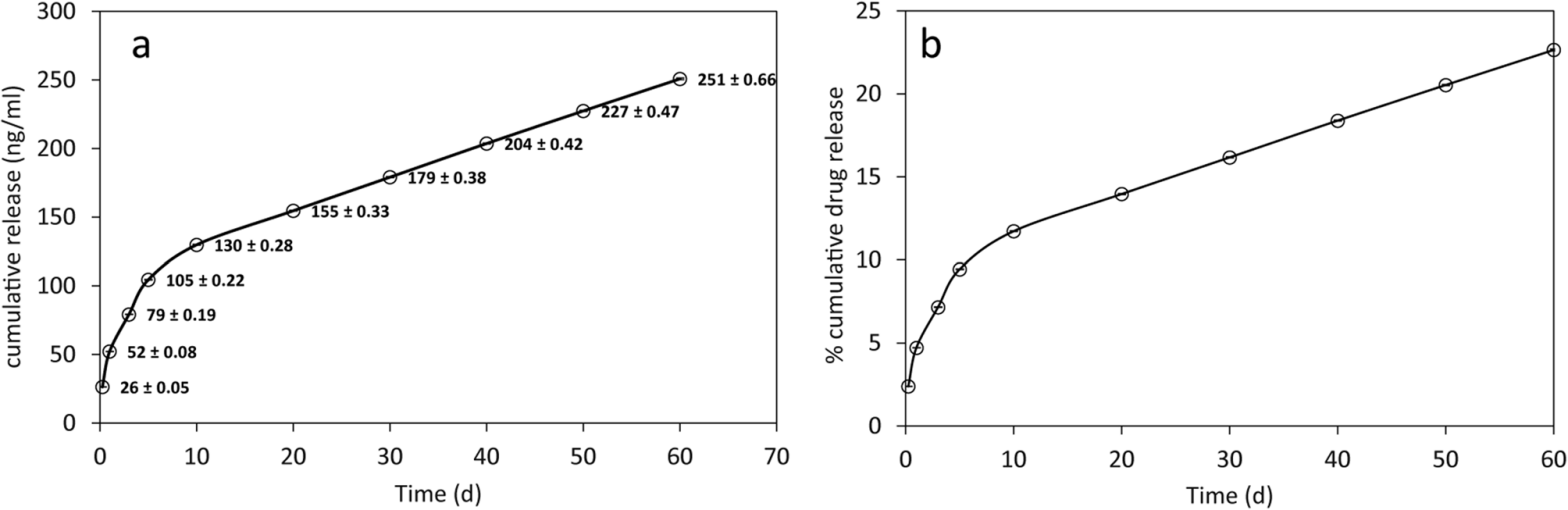
In vitro drug release curves for nerve growth factor beta (NGF-β) release from BPC implants. (**a**) At each sampling time point, the mean cumulative concentration of NGF-β detected in 200 µl of sample is plotted, representing the mean of measurements from six independent samples. Standard deviation error bars were smaller than the circles used to plot the data points and the standard deviations are instead indicated alongside the plotted data points. (**b**) Based on the total loaded amount of NGF-β, the cumulative percentage drug release curve was calculated.

The total amount of NGF-β loaded into each BPC implant sample for in vitro testing was 1107 ng. The cumulative release curve for NGF-β during the 60-day in vitro test is given in Fig. 12, expressed in terms of cumulative concentration of NGF-β released in 200 µl samples, and cumulative percentage release. After 60 days, approximately 23% of NGF-β was released from the implants. The cumulative release curve was linear after an initial exponential release lasting 10 days, indicating a gradual linear release of the incorporated NGF-β thereafter.

## Discussion

We present a porous, highly transparent hydrogel manufactured from medical-grade porcine collagen, having the additional capability of regenerative drug loading and delivery in vitro. The BPC hydrogel exhibited a uniform nanostructure with high optical transparency in the visible wavelength range, with transparency thought to reflect the regularity of arrangement of collagen fibers at the nanoscale^20^. While lower transmittance of the human cornea in the ultraviolet range can be beneficial for protecting the eye against damaging radiation, much of the ultraviolet absorption is attributed to the corneal epithelium, which was not present on BPC materials during transmission measurements (but is present in vivo postoperatively). The BPC was mechanically stronger than an earlier version of porcine-based implants we previously reported^15^, with improved ultimate stress, stiffness, and toughness; however, these parameters were still reduced relative to the human cornea^21^. As a result, the mechanical tension of directly suturing the BPC into porcine eyes ex vivo resulted in micro-fissures in the material. For this reason, implantation methods avoiding direct suturing of the BPC were used. Subcutaneous implantation in rats (with suturing of overlying skin only) revealed an intact BPC eight weeks post-implantation, with recipient α-SMA-expressing fibroblasts accumulating at the implant-host interface and producing new type III collagen, as a wound healing response. This host healing response was non-inflammatory, but consisted of an initial loss of adjacent β-III-tubulin-expressing nerves, that eventually repopulated the implant-host interface region. Cell biocompatibility was demonstrated by confluent growth of human corneal epithelial cells on BPC, confirming prior results of cell compatibility of materials with human corneal stromal mesenchymal stem cells^12^.

Implantation of BPC into the rabbit corneal stroma in vivo was achieved by two methods, the first using overlying sutures in an ALK procedure, to avoid suture-induced tension on the BPC. We previously reported a similar method for implanting human collagen-based hydrogel implants in a clinical study^9^; however, the method triggered significant implant thinning in some cases. Here, the ALK method resulted in thickened rabbit corneas in autografts (mean central thickness of 490 µm versus 380 µm for the native cornea), whereas thickness was maintained in BPC-implanted corneas (mean of 406 µm). The reason for increased thickness in autografts is unclear, but may be related to early postoperative swelling (observed by OCT, Fig. 6c) and new collagen production postoperatively, as diffuse type III collagen staining was noted in the corneal stroma and a low level of clinical haze was observed clinically at 6 months (Fig. 7b). Although in the ALK procedure with BPC implants a normal corneal thickness was maintained 6 months postoperatively, fibroblast-mediated production of compacted, light-scattering type III collagen was apparent throughout the implanted region, with the original implanted material being otherwise indistinguishable from the native corneal stroma. The result was an increased corneal haze/opacity clinically. Gradual biomaterial degradation may have promoted the new collagen production; however, direct observation of this process would require more frequent postoperative observation. These findings recapitulate observations after implantation of human collagen-based hydrogels by ALK, where implant thinning and local corneal haze were observed clinically during the early postoperative period, thereafter stabilizing^9,11^. Thus, there remains a need for a surgical implantation approach that preserves the biomaterial for longer periods of time without inducing implant thinning or scar formation.

The suboptimal results of ALK implantation of biomaterials in the rabbit model led us to evaluate a second alternative, implantation via a new hybrid Flap-ALK model. This is a further development of the intra-stromal implantation model (FLISK) which we previously reported^12^, with important differences being the ability to implant larger (7 mm vs. 3 mm) and thicker (> 250 µm vs. 150 µm) hydrogels for diseased corneal stroma replacement, and a controlled implantation procedure that can be achieved manually using a microtome for flap creation when a femtosecond laser is unavailable. These changes were instated to resemble the envisioned clinical use of the procedure in human eyes. Because the flap covers the implant, the BPC material is protected from the sutures and the need for epithelial cell migration and proliferation on the material is minimized. This allows more rapid wound healing, as observed with the fluorescein staining test. The Flap-ALK model resulted in final corneal thicknesses closer to that of the native cornea, with slightly improved mean corneal transparency and slightly reduced neovascularization score. Histologically, the flap and BPC regions were clearly identifiable, with a small number of fibroblasts visible at implant interfaces, where type III collagen expression was also evident. Notably, the bulk of the implanted BPC was preserved and histologically unequivocally identifiable as a distinct stromal region with minor host cell infiltration. No CD45+ leukocytes were observed in the Flap-ALK model. This Flap-ALK model represents a new approach to maintain both epithelial and endothelial layers, as well as subbasal nerves while enabling augmentation of the corneal stroma (eg. to treat keratoconus) or partial replacement of the stroma (eg. to treat scarring or dystrophies). The presence of flap-related complications or poor flap adhesion to the underlying material was not observed in this study, as all flaps were adherent (Figs. 9, 10, 11) and no flap loosening was noted while mechanical tension was applied during the suture removal procedure. The possibility of flap-related complications (noted in a small percentage of LASIK cases clinically) is balanced by the less invasive Flap-ALK procedure that preserves surrounding tissue, relative to alternative lamellar or penetrating keratoplasty that removes epithelium, nerves and/or endothelial layers. A possibility with the Flap-ALK procedure is that if required, it can be converted at a later time to a standard ALK or penetrating keratoplasty, should complications and/or stromal thinning arise.

Interestingly, all BPC implants in the Flap-ALK model were populated to some extent by host stromal cells by 6 months postoperatively. This was likely due to the porous nature of the BPC, also evidenced by a limited collagen degradation directly adjacent to cells within the BPC, supporting our previous findings of cell invasion and stromal collagen turnover several months after implantation into rabbit corneas^12^ to achieve true stromal regeneration. This finding is in contrast to a previous recombinant human collagen-based hydrogel study, where host cell migration into the hydrogel was not observed up to four years postoperatively in a human subject^3,11^. Micro-morphology of the cornea was tracked using laser-scanning in vivo confocal microscopy (IVCM). Examinations revealed a stratified epithelium in all implanted groups, consisting of superficial squamous epithelial cells and a dense mosaic of underlying epithelial wing cells resembling the normal corneal anatomy^19^. Similar to the subcutaneous implantation model, both ALK and Flap-ALK methods of implantation involve transection of nerves in the native tissue, resulting in an initial loss of nerves. In both models, however, nerves were observed at the latest postoperative examinations, indicating at least some nerve regeneration. Nerve regeneration has been tracked following biomaterial implantation in animal models^22^ and in humans^11^, and reinnervation of implanted corneas is typically slow, occurring over a time frame of months to years. Here, the BPC supported at least a partial preservation and/or regeneration of subbasal nerves postoperatively, as evidenced by in vivo examination. Although the flap transects most nerves of the subbasal plexus, nerve regeneration is expected to occur postoperatively, as has been documented following LASIK flap creation in human subjects^23^. Additionally, macrophages were observed in all groups 6 months postoperatively. These cells are indicative of a slow implant remodeling process and were also present in allograft-implanted corneas, suggesting their activity is surgically triggered, possibly as part of a normal wound healing response. Notably, the Flap-ALK method did not induce the infiltration of inflammatory dendritic cells into the central cornea, as was observed in the ALK model. While this result deserves further investigation, it suggests that the immunologic challenge of the Flap-ALK model (which preserves native host epithelium) might be reduced relative to the ALK procedure (where host epithelium is removed and must regenerate entirely). Finally, the Flap-ALK model theoretically allows for LASIK-type corrective refractive surgery with BPC inlays and shaping of the cornea to achieve the best refraction for vision, either at the time of initial BPC implantation or later (by re-opening the flap).

Corneal neovascularization limited to the peripheral implanted zone was observed during the first postoperative month in all implanted groups, but gradually reduced over time in all groups except in the BPC ALK group. Neovascularization in both Flap-ALK groups and the location of vessels relative to sutures indicated that vessel growth was initially triggered by the presence of surgical sutures in the cornea. Postoperative steroids were generally discontinued during the second postoperative week; however, a longer duration of postoperative steroids (i.e., until suture removal) would likely have had a stronger suppressive effect on new vessel growth. Nevertheless, the minimal vessels observed did not enter the central corneal region, where a detrimental effect on vision would be expected. In a prior study^12^, suture-free intra-stromal implantation of BPC was not accompanied by a neovascular response, providing further evidence that the surgical method and postoperative medication regimen, but not the BPC material itself, influence this parameter.

In addition to use of the BPC as a cell-free stromal replacement passively allowing endogenous regeneration of corneal epithelium, nerves, and stroma, we tested for the first time the ability of this porous biomaterial to serve as a reservoir for delivery of a regenerative drug. Exemplified by the incorporation of recombinant nerve growth factor, a substance recently approved for clinical use for regeneration of the corneal neurotrophic environment^24,25^, the BPC achieved sustained drug release in vitro for up to two months. Based on the amount of drug remaining in the implant, it can be linearly extrapolated that drug delivery would continue for at least 6 months, to provide a sustained regenerative local environment on physiologic time frames for corneal nerve regeneration^23,26^. Sustained delivery of drugs directly within the corneal stroma could circumvent problems of patient compliance, the typical short residence time of ophthalmic drugs on the ocular surface and the low efficacy of topical drug delivery across an intact epithelial barrier^27^. Further studies, using the intra-stromal implantation method described here, would be required to evaluate the feasibility of biomaterial-assisted intra-stromal drug delivery in vivo.

## Conclusions

In summary, we present a transparent, porous hydrogel biomaterial, BPC, manufactured from a medical-grade porcine collagen. BPC has the potential for scalability, to address the global demand for suitable tissue to treat corneal blindness. In addition to favorable optical and mechanical properties, the BPC was compatible with cell ingrowth and overgrowth, and can be implanted into the corneal stroma using tools and methods mimicking those used in human corneal surgery. Using a new Flap-ALK implantation model, a large-diameter BPC implant can replace a portion of the native corneal stroma, with rapid wound healing promoting a stable and transparent cornea. In vivo, the BPC permitted host stromal cell migration, epithelial and nerve regeneration while maintaining corneal shape and thickness during a 6-month postoperative period. The favorable properties of the BPC and the proposed implantation method suggest the translational potential of this approach, and the future possibility to combine corneal stromal replacement therapy with active delivery of pro-regenerative drugs.
